# Supplementary material for: Mitochondrially targeted tamoxifen alleviates markers of obesity and type 2 diabetes mellitus in mice
Source: Nat Commun. 2022 Apr 6;13:1866. doi: 10.1038/s41467-022-29486-z (PMC8987092; doi:10.1038/s41467-022-29486-z)
Supplement: Supplementary file 1 — Supplementary Information [file 41467_2022_29486_MOESM1_ESM.pdf]

Supplementary Material

Materials and methods

Patients

To obtain samples from lean subjects and patients with obesity (BMI > 35 kg/m2) with or without type 2 diabetes patients of at least 50 years of age were recruited. Subcutaneous and visceral adipose tissue samples from lean subjects (female=7, male=1) were taken during elective abdominal surgeries (cholecystectomy). Subcutaneous and visceral adipose tissue samples from patients with obesity with diabetes (female=8, male=2; BMI= 44,65±10,69 kg/m^2; glycemia=8,71±2,14 mmol/L) or without diabetes (female=4, male=4; BMI= 43,03±10,63 kg/m^2; glycemia=4,94±0,65 mmol/L) were taken during bariatric surgery (sleeve gastrectomy). Samples were obtained from patients undergoing surgery in Institute for Clinical and Experimental Medicine (IKEM, Prague, Czech Republic). All participants signed written informed consent prior to the enrolment into the study. Since the samples were taken as a part of a planned operation, the patients were not compensated. The study was approved by Human Ethics Review Board of IKEM.

Indirect calorimetry

Indirect calorimetry was performed in the PhenoMaster system (TSE Systems, Bad Homburg, Germany). Food and water was provided ad libitum, SD or HFD were used. All mice in the cohort were weighed before the start of the evaluation. Individually housed mice (n=8) were acclimatised to the indirect calorimetry system (23 °C, 55 % relative humidity, light cycle of 12 h) for about 24 h. Daily energy expenditure (EE), respiratory exchange ratio (RER), locomotor activity, heat production, CO_2, O_2 and food and water intake were read every 15 min for 24 h. The data were evaluated using TSE PhenoMaster v.7.1.2. After 48 h of data collection, the experiment was stopped, and the mice were weighed and placed in their original cages.

Electrocardiography (ECG)

Electrocardiographs were recorded non-invasively in conscious mice using the ECGenie recording system (Mouse Specifics, Framingham, MA. USA). Briefly, mice were placed on a platform fitted with ECG footplate electrodes (#MSI001) and allowed to adapt for ~10 min.

After the acclimation period, ECG signals were recorded for 5 min. Data acquisition was carried out using the program LabChart 8 (ADInstruments, Oxford, United Kingdom). Analysis of individual ECG signals was then performed using the EzCG analysis software (MouseSpecifics, Framingham, MA, USA). At least 50 ECG signals for each mouse per time point were analyzed.

Protein digestion, nLC-MS and data analysis

Tissues were homogenised and lysed by boiling at 95 °C for 10 min in 100 mM triethylammonium bicarbonate (TEAB) containing 2 % sodium deoxycholate, 40 mM chloroacetamide, 10 mM Tris(2-carboxyethyl)phosphine (TCEP) and further sonicated (Sonopuls Mini 20, MS 1.5, Bandelin, Electronic, Berlin, Germany). Protein concentration was determined using the BCA protein assay kit (Thermo), and 30 μg of protein per sample was used for MS sample preparation.

Samples were further processed using SP3 beads [1]. Briefly, 5 µl of SP3 beads was added to 30 µg of protein in lysis buffer and filled to 50 µL with 100 mM TEAB. Protein binding was induced by addition of ethanol to 60 % (v/v) final concentration. Samples were mixed and incubated for 5 min at RT. The tubes were then placed into a magnetic rack and the unbound supernatant discarded. Beads were subsequently washed two times with 180 µL of 80 % ethanol. Samples were digested with trypsine (trypsin/protein ratio, 1/30) and reconstituted in 100 mM TEAB at 37 °C overnight. Samples were then acidified with trifluoroacetic acid (TFA) to 1 % final concentration and peptides were desalted using in-house made stage tips packed with C18 disks (Empore, Oxford, PA, USA) [2].

Nano Reversed phase columns (EASY-Spray column, 50 cm x 75 µm ID, PepMap C18, 2 µm particles, 100 Å pore size) were used for LC/MS analysis. Mobile phase buffer A was composed of water and 0.1% formic acid. Mobile phase B was composed of acetonitrile and 0.1% formic acid. Samples were loaded onto the trap column (C18 PepMap100, 5 μm particle size, 300 μm x 5 mm, Thermo Scientific) for 4 min at 18 μl/min loading buffer was composed of water, 2% acetonitrile and 0.1% trifluoroacetic acid. Peptides were eluted with Mobile phase B gradient from 4% to 35% B in 120 min. Eluted peptide cations were converted to gas-phase ions by electrospray ionization and analyzed on a Thermo Orbitrap Fusion (Q-OT-qIT, Thermo Scientific). Survey scans of peptide precursors from 350 to 1400 m/z were performed in the Orbitrap at 120K resolution (at 200 m/z) with a $5x10^5$ ion count target. Tandem MS was

performed by isolation at 1.5 Th with the quadrupole, HCD fragmentation with normalized collision energy of 30, and rapid scan MS analysis in the ion trap. The MS2 ion count target was set to 10^4 and the maximum injection time was 35 ms. Only those precursors with charge state 2–6 were sampled for MS2. The dynamic exclusion duration was set to 45 s with a 10 ppm tolerance around the selected precursor and its isotopes. Monoisotopic precursor selection was turned on. The instrument was run in top speed mode with 2 s cycles [3].

All data were analyzed and quantified with the MaxQuant software (version 1.6.3.4) [4]. The false discovery rate (FDR) was set to 1 % for both proteins and peptides, and we specified a minimum peptide length of seven amino acids. The Andromeda search engine was used for the MS/MS spectra search against the *Mus musculus* dabase (downloaded from Uniprot on July 2019, containing 22 267 entries). Enzyme specificity was set as C-terminal to Arg and Lys, also allowing cleavage at proline bonds and a maximum of two missed cleavages. Dithiomethylation of cysteine was selected as fixed modification and N- terminal protein acetylation and methionine oxidation as variable modifications. The “match between runs” feature of MaxQuant was used to transfer identifications to other LC-MS/MS runs based on their masses and retention time (maximum deviation 0.7 min), and this was also used in quantification experiments. Quantifications were performed with the label-free algorithm in MaxQuant [5]. Data analysis was performed using the Perseus 1.6.1.3 software [6].

Chemicals, antibodies and primers

The following chemicals were used in the study:

MitoTam		Rohlenova et al., 2017
Tamoxifen	T5648	Sigma

For immunofluorescent staining, the following antibodies were used:

anti-TOM20	EPR15581-54	Abcam

Antibody was diluted 1:100 in PBS. Secondary antibody Alexa 488 goat anti-rabbit (cat.n. A11034) was purchased from ThermoFisher and diluted 1:1,000 in PBS. Primary antibody was

tested on 3T3-L1 cells without Alexa488 labeled secondary antibody using indirect immunofluorescence method to avoid its nonspecific signal. To check its specific binding on mitochondria, colocalization with MitoTracker was tested.

The following primers for qRT-PCR were purchased from Sigma:

ACTB_F	CCAACCGCGAGAAGATGA	mCD11c_F	TGCCAGGATGACCTTAGTGTCG
ACTB_R	CCAGAGGCGTACAGGGATAG	mCD11c_R	CAGAGTGACTGTGGTTCCGTAG
mACTB_F	CATTGCTGACAGGATGCAGAAGG	mCEBPa_F	GCAAAGCCAAGAAGTCGGTGGA
mACTB_R	TGCTGGAAGGTGGACAGTGAGG	mCEBPa_R	CCTTCTGTTGCGTCTCCACGTT
mAdipoq_F	TGTTCCTCTTAATCCTGCCC	mHIF1α_F	CCTGCACTGAATCAAGAGGTTGC
mAdipoq_R	CCAACCTGCACAAGTTCCCT	mHIF1α_R	CCATCAGAAGGACTTGCTGGCT
mANT2_F	ACACGGTTCGCCGTCGTATGAT	mIL-8_F	CTCTATTCTGCCAGATGCTGTCC
mANT2_R	AAAGCCTTGCTCCCTTCATCGC	mIL-8_R	ACAAGGCTCAGCAGAGTCACCA
mArf_F (p19)	GCCGCACCGGAATCCTGACGC	mINS1_F	CGTGGCTTCTTCTACACACCCA
mArf_R (p19)	TTGAGCAGAAGAGCTGCTACGT	mINS1_R	TGCAGCACTGATCCACAATGCC
mB2M1_F	ATGGGAAGCCGAACATACTG	mINS2_F	CGTGGCTTCTTCTACACACCCA
mB2M1_R	CAGTCTCAGTGGGGGTGAAT	mINS2_R	TCCAGTGCCAAGGTCTGAAGGT
CDKN1A_F (p21)	TCACTGTCTTGTACCCTTGTGC	mLEP_F	GCAGTGCCTATCCAGAAAGTCC
CDKN1A_R (p21)	GGCGTTTGGAGTGGTAGAAA	mLEP_R	GGAATGAAGTCCAAGCCAGTGAC
mCDKN1A_F (p21)	TCGCTGTCTTGCACTCTGGTGT	mMCP-1_F (CCL2)	GCTACAAGAGGATCACCAGCAG
mCDKN1A_R (p21)	CCAATCTGCGCTTGGAGTGATAG	mMCP-1_R (CCL2)	GTCTGGACCCATTCCTTCTTGG
mCDKN2A_F (p16)	TGTTGAGGCTAGAGAGGATCTTG	mTNFa_F	GCCTCTTCTCATTCCTGCTTG
mCDKN2A_R (p16)	CGAATCTGCACCGTAGTTGAGC	mTNFa_R	CTGATGAGAGGGAGGCCATT

References:

1 Hughes, C. S. *et al.* Single-pot, solid-phase-enhanced sample preparation for proteomics experiments. *Nat Protoc* **14**, 68-85, (2019).

2 Rappsilber, J., Mann, M. & Ishihama, Y. Protocol for micro-purification, enrichment, pre-fractionation and storage of peptides for proteomics using StageTips. *Nat Protoc* **2**, 1896-1906, (2007).

3 Hebert, A. S. *et al.* The one hour yeast proteome. *Mol Cell Proteomics* **13**, 339-347, (2014).

4 Cox, J. & Mann, M. MaxQuant enables high peptide identification rates, individualized p.p.b.-range mass accuracies and proteome-wide protein quantification. *Nat Biotechnol* **26**, 1367-1372, (2008).
5 Cox, J. *et al.* Accurate proteome-wide label-free quantification by delayed normalization and maximal peptide ratio extraction, termed MaxLFQ. *Mol Cell Proteomics* **13**, 2513-2526, (2014).
6 Tyanova, S. *et al.* The Perseus computational platform for comprehensive analysis of (prote)omics data. *Nat Methods* **13**, 731-740, (2016).

Supplementary Figure 1: Obesity and T2DM are linked to premature senescence.

C57BL/6 mice 2 (young) and 18 month of age (aged) were treated i.p. once per week for a period of 4 weeks with MitoTam (2 mg/kg body weight; MT) dissolved in 4 % EtOH in corn oil or with the vehicle (CO). **(a, b, c)** Expression of *p16*^{Ink4a} and *p19* ^{Ink4d} mRNA in lungs (**a**; p=0.0292), liver (**b**; young+CO vs. aged+CO p=0.03; young+CO vs. aged+MT p=0.047; **a,b** young n=8; aged n=11; aged+MT n=8) and VAT (**c**; n=7) was estimated by qRT-PCR. **(d, e)** Total body weight was measured once per week throughout the experiment, Δ body weight (Δ B.W.) was calculated as a difference between initial and final body weight (**d**; young+CO vs. aged+CO p<0.001; young+CO vs. aged+MT p=0.004) together with visceral adipose tissue (VAT) weighted at the end of the experiment (**e**; p=0.035). **(f)** Food intake was expressed as kcal/day per mouse (young+CO vs. aged+CO p<0.001, young+CO vs. aged+MT at week 1 and 2 p<0.001, young+CO vs. aged+MT at week 3 p=0.046) (**d-f** young n=10; aged, aged+MT n=8). **(g)** Liver from C57BL/6 mice fed SD or HFD for 15 weeks was assessed for SA-β-gal staining, expressed as % of SA-β-gal positive tissue (p=0.0002). **(h-k)** mRNA levels of *p21*^{waf1} (p=0.0007), *MCP-1, TNFα* (p=0.0436) and *IL-8* (p=0.0023) were assessed in liver tissue by qRT-PCR (**g-k** SD n=9; HFD n=10). **(l)** mRNA levels of *p21*^{waf1} in VAT tissue of human control patients, patients with obesity and patients with T2DM were assessed by qRT-PCR (control n=7; Obese n=8; T2DM n=10).

For panels **a-f** and **l** One-way ANOVA, Tukey´s comparison multiple test was used. For panels **g-k** Unpaired t-test was used. Data are expressed as mean ± SEM; *p<0.033; **p<0.002; ***p<0.001. Source data are provided as a Supplementary Source Data file.

Supplementary Figure 2: MitoTam improves metabolic profile and attenuates senescence markers in mice fed high-fat diet.

C57BL/6 male mice fed with SD (standard diet), HFD (high fat diet) or HFD+PF (pair feed high fat diet) were treated with MitoTam (2 mg/kg body weight; MT) dissolved in 4 % ethanol in corn oil or the vehicle (CO) given i.p. twice per week for a period of 4 weeks.

(a) Cumulative food intake expressed in kcal during experiment (HFD+CO n=6; HFD+MT,HFD+PF n=7; HFD+CO vs. HFD+MT p=0.0114). **(b)** Weight of EAT, PRAT and SAT was measured (n=10; EAT, PRAT p<0.001; SAT: SD+CO vs. HFD+CO p<0.001; HFD+CO vs. HFD+MT p=0.006). **(c)** Senescent marker *p21*^{waf1} in EAT (SD+CO,HFD+CO,HFD+MT n=9; SD+MT n=10; p=0.031) and **(d)** *p16*^{Ink4a} (HFD+CO,HFD+PF n=6; HFD+MT n=7), *p21*^{waf1} and

p19 ^{Ink4d} in EAT from PF experiment (HFD+CO n=6, HFD+MT,HFD+PF n=7; p=0.032) were evaluated by qRT-PCR. **(e)** *UCP1* mRNA expression in SAT (SD+CO n=9; SD+MT,HFD+CO,HFD+MT n=10; p=0.022) and **(f)** SAT from PF experiment was evaluated by qRT-PCR (HFD+CO, HFD+MT n=6, HFD+CO=7; p=0.037). **(g, h)** Blood glucose during oral glucose tolerance test (oGTT) was assessed at time points as indicated (**g;** n=10; HFD+CO vs. HFD+MT: 0 min p=0.004; 15, 30, 60 min p<0.001; 120 min p=0.004; 180 min p=0.0086. **h;** HFD+CO n=6, HFD+MT, HFD+PF n=7; HFD+CO vs. HFD+MT: 15 min p=0.024; 30, 60 min p<0.001; HFD+CO vs. HFD+PF 30 min p=0.049; 60 min p<0.001; HFD+MT vs. HFD+PF 60 min p=0.049). **(i)** Level of glucose in feces measured by mass spectrometry (AU; auxiliary unit) (HFD+CO n=5; HFD+MT,HFD+PF n=7; p=0.043). **(j)** Expression of *leptin* mRNA in EAT (n=10, p<0.001) and **(k)** insulin 1 (*INS1*) and insulin 2 (*INS2*) mRNA in pancreas (n=10) was evaluated by qRT-PCR. **(l)** TNFα and MCP1 protein levels in plasma were assessed (SD+CO,HFD+MT n=9; SD+MT,HFD+CO n=10). **(m)** Accumulation of damage in kidneys (tubular necrosis) was detected in histological samples stained with haematoxylin and eosin.

For panels **a,g,h** Two-Way ANOVA, Bonferroni´s multiple comparisons test was used. For panels **b-f** and **i-l** One-Way ANOVA, Tukey´s comparison multiple test was used. All data are expressed as mean ± SEM; *p<0.033; **p<0.002; ***p<0.001. Source data are provided as a Supplementary Source Data file.

Supplementary Figure 3: MitoTam is superior to tamoxifen in improvement of diabetic parameters.

HFD-fed C57BL/6 mice (16 month old males) were divided into three groups: HFD+CO (corn oil); HFD+MT (MitoTam); HFD+TX (tamoxifen); the animals were treated with MitoTam or tamoxifen (2 mg/kg body weight) dissolved in 4 % ethanol in corn oil or the vehicle given i.p. twice per week for a period of 4 weeks.

(a) Weight of EAT, PRAT and SAT was measured (n=6; HFD+CO vs. HFD+MT p=0.0034, HFD+CO vs. HFD+TX p=0.0052). **(b)** Blood glucose during oral glucose tolerance test (oGTT) was assessed at time points shown (HFD+CO vs. HFD+MT: 0 min p=0.008; 15 min p=0.01; 60 min p=0.004; 120 and 180 min p<0.001). **(c)** Levels of fasting TAG were evaluated in plasma. **(d)** TAG levels in the liver were assessed (HFD+CO vs. HFD+MT p=0.0005; HFD+CO vs. HFD+TX

p=0.0006; **b-d** HFD+CO, HFD+TX n=7, HFD+MT n=8). **(e)** *p21*^{waf1} mRNA levels in the liver were evaluated by qRT-PCR (HFD+CO, HFD+TX n=6; HFD+MT n=4).

For panel **a** and **c-e** One way ANOVA, Tukey´s comparison multiple test was used (except figure b). For panel **b** Two-way ANOVA, Tukey´s multiple comparisons test was used. Data are expressed as mean ± SEM; *p<0.033; **p<0.002; ***p<0.001. Source data are provided as a Supplementary Source Data file.

Supplementary Figure 4: Prolonged effect of MitoTam on mice with impaired metabolism.

C57BL/6 mice (6 month old males) fed SD (standard diet) or HFD (high fat diet) were treated i.p. twice per week for a period of 4 weeks with MitoTam (2 mg/kg of body weight; MT) dissolved in 4 % EtOH in corn oil or with the vehicle (CO). **(a)** Intraperitoneal glucose tolerance test (IPG) was performed at the end and one month after the end of the treatment at time points indicated (after treatment: SD+CO, SD+MT n=7; HFD+CO, HFD+MT n=8; 1 m after treatment SD+CO, SD+MT n=7; HFD+CO n=8; HFD+MT=6). **(b)** Area of total fat in core body was determined before and after treatment (representative images). Each image represents overlap of two scans (before treatment and at the end of treatment, scans were superimposed based on the position of mouse backbone and ribs), purple color represents overlap of adipose tissue present in the mouse during both scans. Blue color represents adipose tissue detected only during scanning before treatment, red color reveals adipose tissue detected only immediately after the treatment. **(c)** The levels of insulin 1 (*INS1*) and insulin 2 (*INS2*) mRNA in the pancreas and **(d)** *leptin* mRNA in VAT (**d**; SD+CO vs. HFD+CO p=0.002; HFD+CO vs. HFD+MT p=0.041) were evaluated by qRT-PCR. **(e)** Levels of triglycerides (TAG) in the liver were evaluated (SD+CO vs. HFD+CO p<0.001, HFD+CO vs. HFD+MT p=0.009). **(f)** Organs were weighted at the end of experiment. **c-f**; SD+CO, SD+MT n=7; HFD+CO n=8; HFD+MT=6. One-way ANOVA, Tukey´s comparison multiple test was used. Data are expressed as mean ± SEM; *p<0.033; **p<0.002; ***p<0.001. Source data are provided as a Supplementary Source Data file.

Supplementary Figure 5: Effect of MitoTam on energy metabolism.

Mice were studied in an automated indirect calorimetry system before, at the end, and one month after the end of MitoTam treatment, with measurements of total energy expenditure(TEE) (**a,b**; SD+CO vs. HFD+MT and SD+CO vs. HFD+CO p<0.0001, HFD+CO vs. HFD+MT p=0.0287,

HFD+CO vs. HFD+MT p=0.0114), respiratory exchange ratio (RER) (**c,d**; SD+CO vs. HFD+CO p=0.0056, HFD+CO vs. HFD+MT p=0.0014, SD+CO vs. HFD+MT p<0.0001), food intake(FI) **(e,f)**, and physical activity(PA) (**g,h**; p=0.0089). Mice were acclimated to the system for one day and then studied for 24h starting at 06:00 (onset of light). Data on **a,c,e,g** are group means using 30 min bins; for visual clarity, error bars are omitted; dark phase periods are indicated as black rectangles on TEE graph (**a**). Data on **b,d,f,h** show 24 h mean of particular parameter; (**i**) To study how TEE depends on body weight (BW), TEE and BW were analyzed by linear regression in all studied groups. Parameters of linear regression are: **before treatment,** using all points: TEE=0.148*BW+3.396, R^2=0.78, p<0.0001; SD+CO: TEE=0.280*BW-0.235, R^2=0.47, p=0.061; SD+MT: TEE=0.041*BW+8.65, R^2=0.025, p=0.71; HFD+CO: TEE=0.236*BW+0.831, R^2=0.63, p=0.019; HFD+MT: TEE=-0.0784*BW+14.3, R^2=0.023, p=0.72; **end of treatment,** using all points: TEE=0.135*BW+3.611, R^2=0.68, p<0.0001; SD+CO: TEE=-0.155*BW+13.0, R^2=0.24, p=0.27; SD+MT: TEE=0.149*BW+2.99, R^2=0.026, p=0.73; HFD+CO: TEE=0.313*BW–5.39, R^2=0.81, p=0.0023; HFD+MT: TEE=0.087*BW+5.37, R^2=0.084, p=0.58, **1 month after treatment,** using all points: TEE=0.101*BW+4.99, R^2=0.43, p=0.001; SD+CO: TEE=0.234*BW+1.27, R^2=0.49, p=0.078; SD+MT: TEE=0.0866*BW+5.25, R^2=0.017, p=0.83; HFD+CO: TEE=0.022*BW+9.24, R^2=0.16, p=0.44; HFD+MT: TEE=0.133*BW+2.50, R^2=0.10, p=0.68. (**a-i**; Before treatment n=8; End of treatment SD+CO,SD+MT n=7; HFD+CO n=8, HFD+MT n=6; 1 m after treatment SD+CO n=7; SD+MT n=5; HFD+CO n=6; HFD+MT n=4). (**j,k**) For each animal, heart intervals and amplitudes were evaluated from continuous ECG recordings at the end and one month after the end of treatment, and total power (**j**; End of treatment SD+CO n=5; SD+MT n=7; HFD+CO n=8; HFD+MT n=7; 1 m after treatment n=7 and heart rate variability (HRV) (**k**; End of treatment SD+CO n=6; SD+MT n=7; HFD+CO n=8; HFD+MT n=7; 1 m after treatment n=7) were assessed. One-way ANOVA, Tukey´s comparison multiple test was used. Data are expressed as mean ±SEM.*p<0.033;**p<0.002;***p<0.0002,****p<0.0001. Source data are provided as a Supplementary Source Data file.

Supplementary Figure 6: MitoTam inhibits differentiation of adipocytes.

3T3-L1 pre-adipocytes were differentiated into mature adipocytes (ADP) while treated with MitoTam (MT) or tamoxifen (TX) during the differentiation process at time points and doses as indicated. **(a,b)** Intracellular lipid accumulation was quantified using Oil Red O in the cells treated

with MitoTam (**a**, n=9; p<0.001; 3T3-L1 vs. 0.16μM MT6-9 p=0.03) or tamoxifen (**b**, n=9; p<0.001); **(c,d)** Representative images of Oil Red O staining of cells treated with MitoTam (**c**) or tamoxifen (**d**). The bar indicates 200 μm. **(e)** qRT-PCR quantification of transcripts of *Pparγ* (HFD+CO, HFD+MT n=6; HFD+TX n=5; HFD+CO vs. HFD+MT p=0.008; HFD+MT vs. HFD+TX p=0.04) and **(f)** *Cebpα* (n=6; HFD+CO vs. HFD+MT and HFD+MT vs. HFD+TX p=0.03) in VAT is shown. **(g)** Proteomic analysis of 3T3-L1 cells treated with MitoTam (MT, 0.32 μM) or tamoxifen (TX, 0.32 μM) in early and late stages of differentiation (n=3). **(h)** Detection of mitochondrial membrane potential by FACS using TMRM staining. CCCP was used as a positive control (basal n=9; CCCP n=8; ADP vs. MT 0-2 p=0.004). **(i)** 3T3-L1 cell death was assessed by flow cytometry using annexin-V/Hoechst staining (n=4; ADP vs. 3T3-L1 p=0.007; ADP vs. MT 0-2 p=0.008). **(j)** qRT-PCR quantification of *ANT2* levels in treated 3T3-L1 cells (MT, 0.32 μM; TX, 0.32 μM) (3T3-L1, ADP, MT 0-2, MT 2-9 n=9; TX 0-2, TX 2-9 n=6; ADP vs. 3T3-L1 p<0.001; ADP vs. MT 0-2 p<0.001; ADP vs. MT 2-9 p=0.001) or **(k)** *ANT2* (HFD+CO n=6; HFD+MT n=5; HFD+TX n=6; HFD+CO vs. HFD+MT p=0.02; HFD+MT vs. HFD+TX p=0.01) and **(l)** *Hif1α* (n=6; HFD+CO vs. HFD+MT p=0.02; HFD+MT vs. HFD+TX p=0.004) in VAT. **(m)** Representative images of SA-β-gal positivity in treated (MT, 0.32 μM; TX, 0.32 μM) or control 3T3-L1 cells are shown. The bar represents 200 μm. The results are from at least two or three independent experiments. For panels **a,b,i,j** One-way ANOVA, Dunnett's multiple comparisons test was used. For panels **e,f,k,l** One-way ANOVA, Tukey´s comparison multiple test was used. For panel **h** Two-way ANOVA, Dunnett's multiple comparisons test was used. Data in all experiments are expressed as mean ± SD;*p<0.033;**p<0.002;***p<0.001. Source data are provided as a Supplementary Source Data file.

Supplementary Figure 7: Mitokines are not essential for the effect of MitoTam.

C57BL/6 male mice fed with SD (standard diet) or HFD (high fat diet) were treated with MitoTam (2 mg/kg body weight; MT) dissolved in 4 % ethanol in corn oil or the vehicle (CO) given i.p. twice per week for a period of 4 weeks.

(a) *FGF21* mRNA (p<0.001) and **(b)** *GDF15* mRNA levels in the liver were evaluated by qRT-PCR. **(c)** *FGF21* mRNA (SD+CO vs. HFD+CO p<0.001; SD+MT vs. HFD+MT p=0.036) and

(d) *GDF15* mRNA (p=0.008) levels in the SAT were evaluated by qRT-PCR. **(e)** Level of FGF21 in plasma was evaluated (p<0.001). (**a-e** n=10).

For panel **a-d** One-way ANOVA, Tukey´s comparison multiple test was used. For panel **e** Two-way ANOVA, Tukey´s multiple comparisons test was used. Data are expressed as mean ± SEM; *p<0.033; **p<0.002; ***p<0.001. Source data are provided as a Supplementary Source Data file.

Supplementary Figure 1

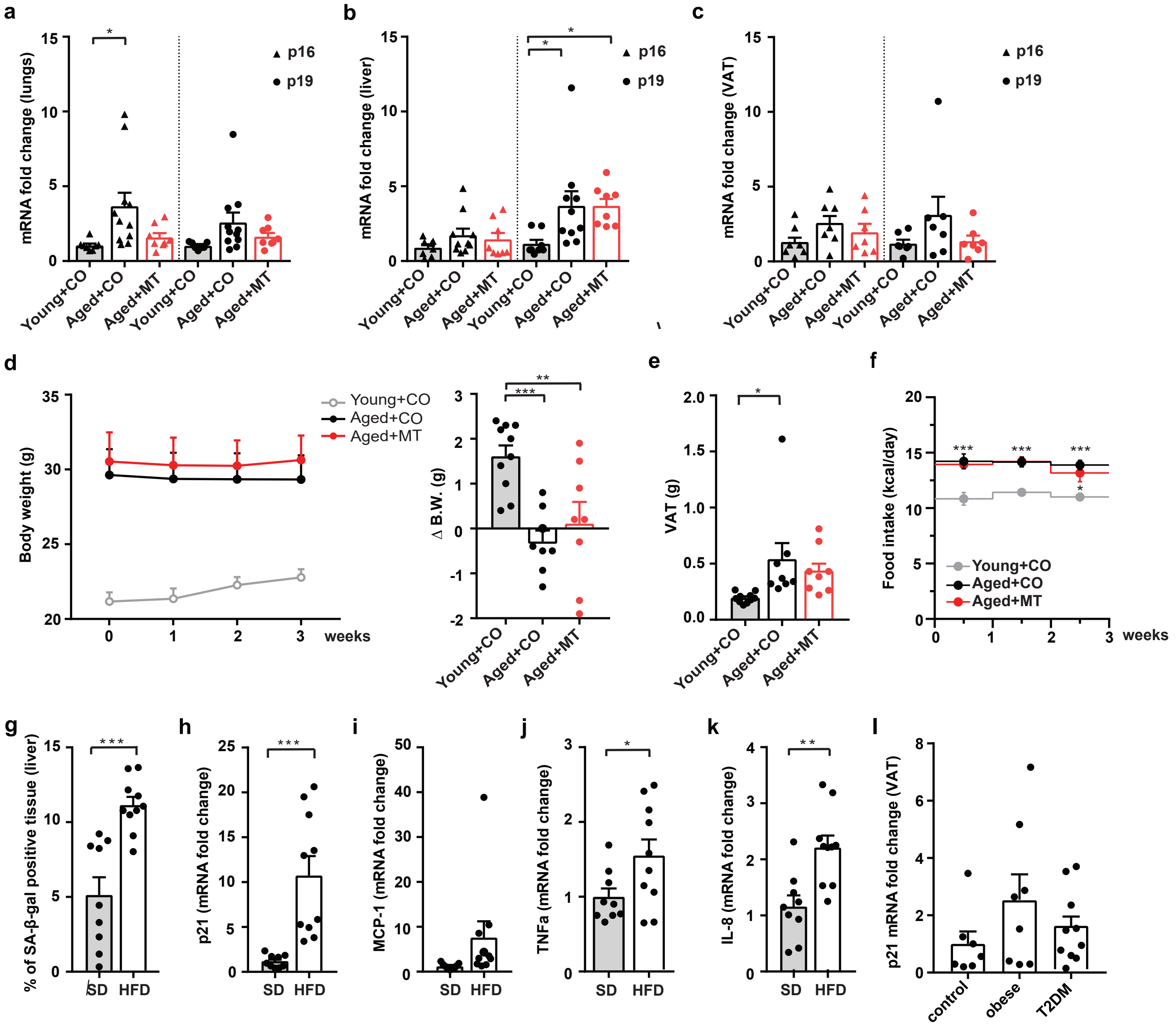

Supplementary figure 2

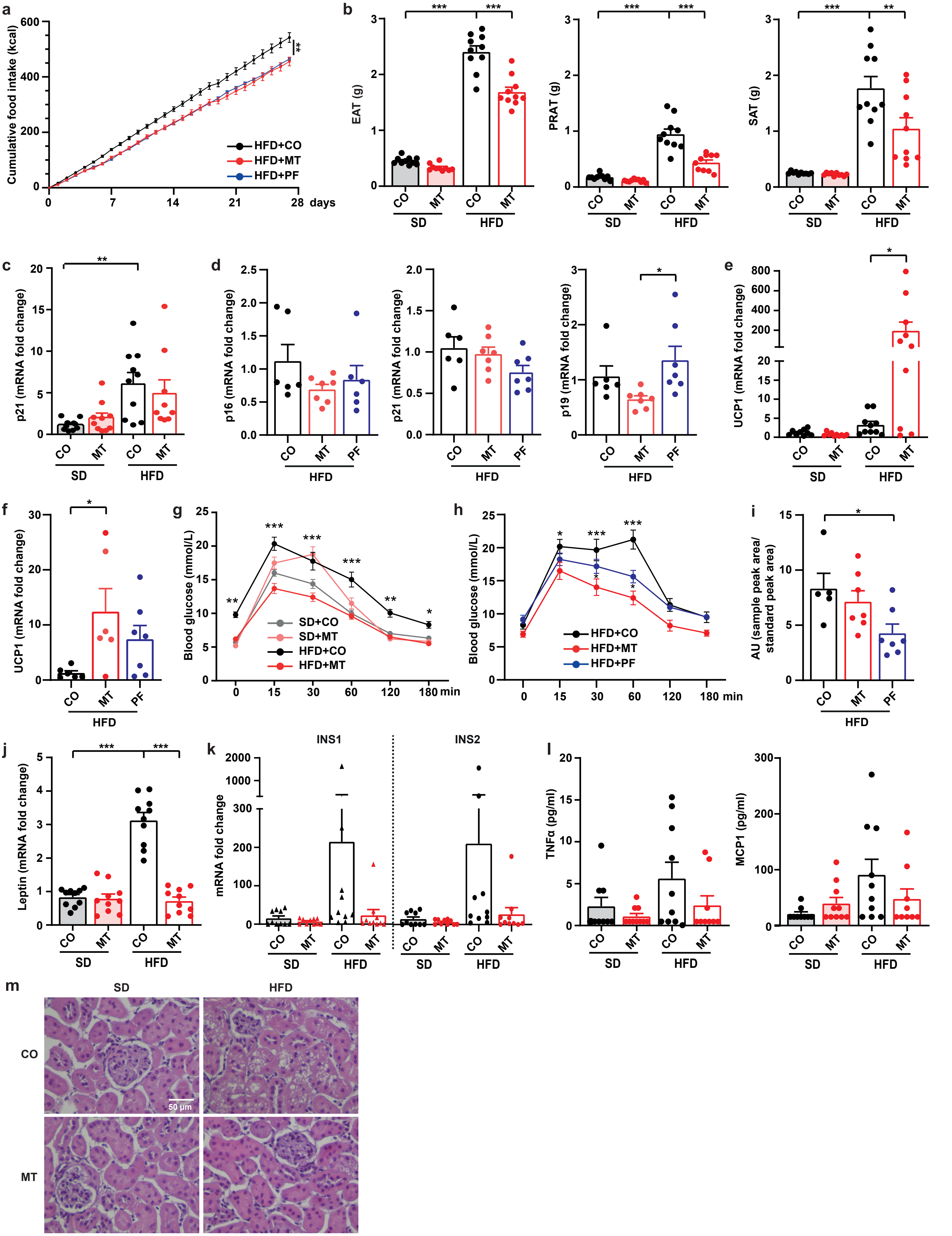

Supplementary figure 3

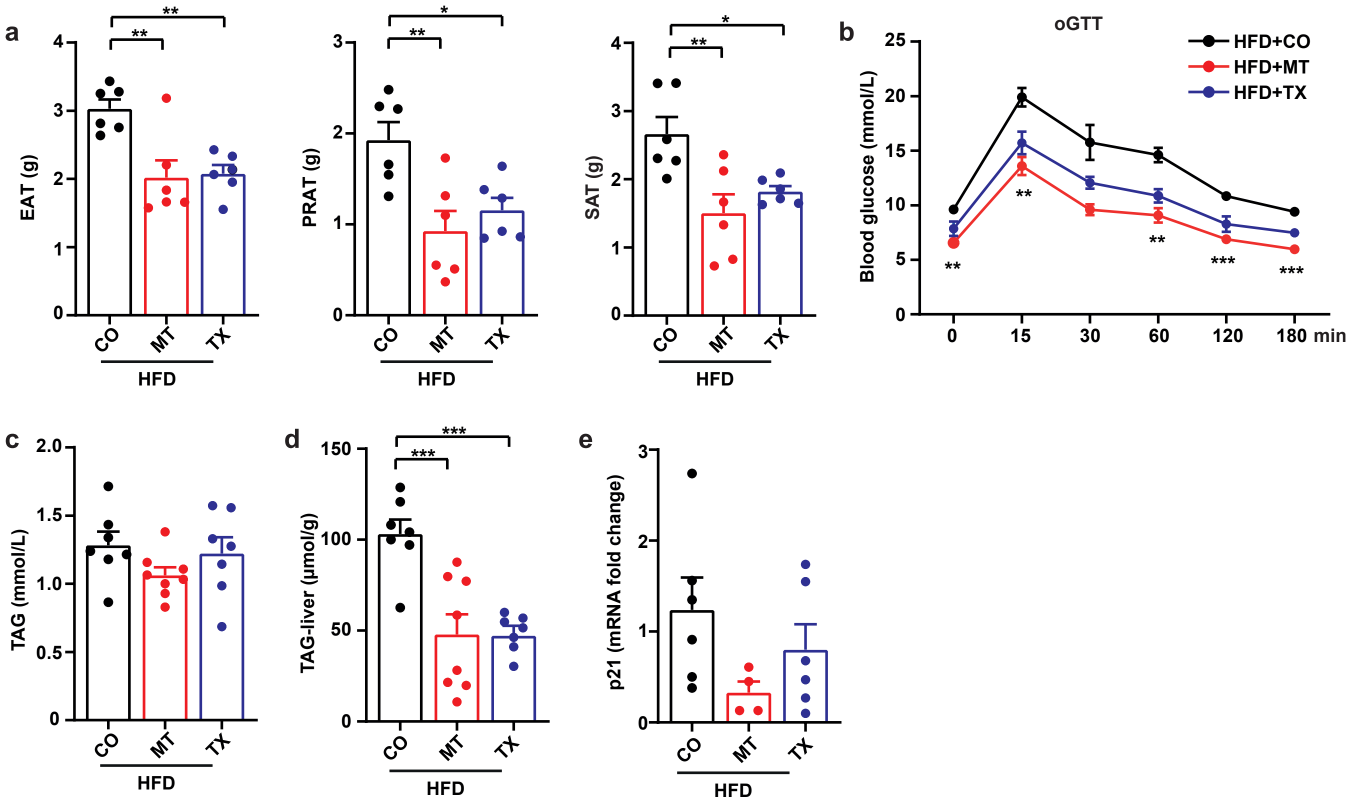

Suppl. Figure 4

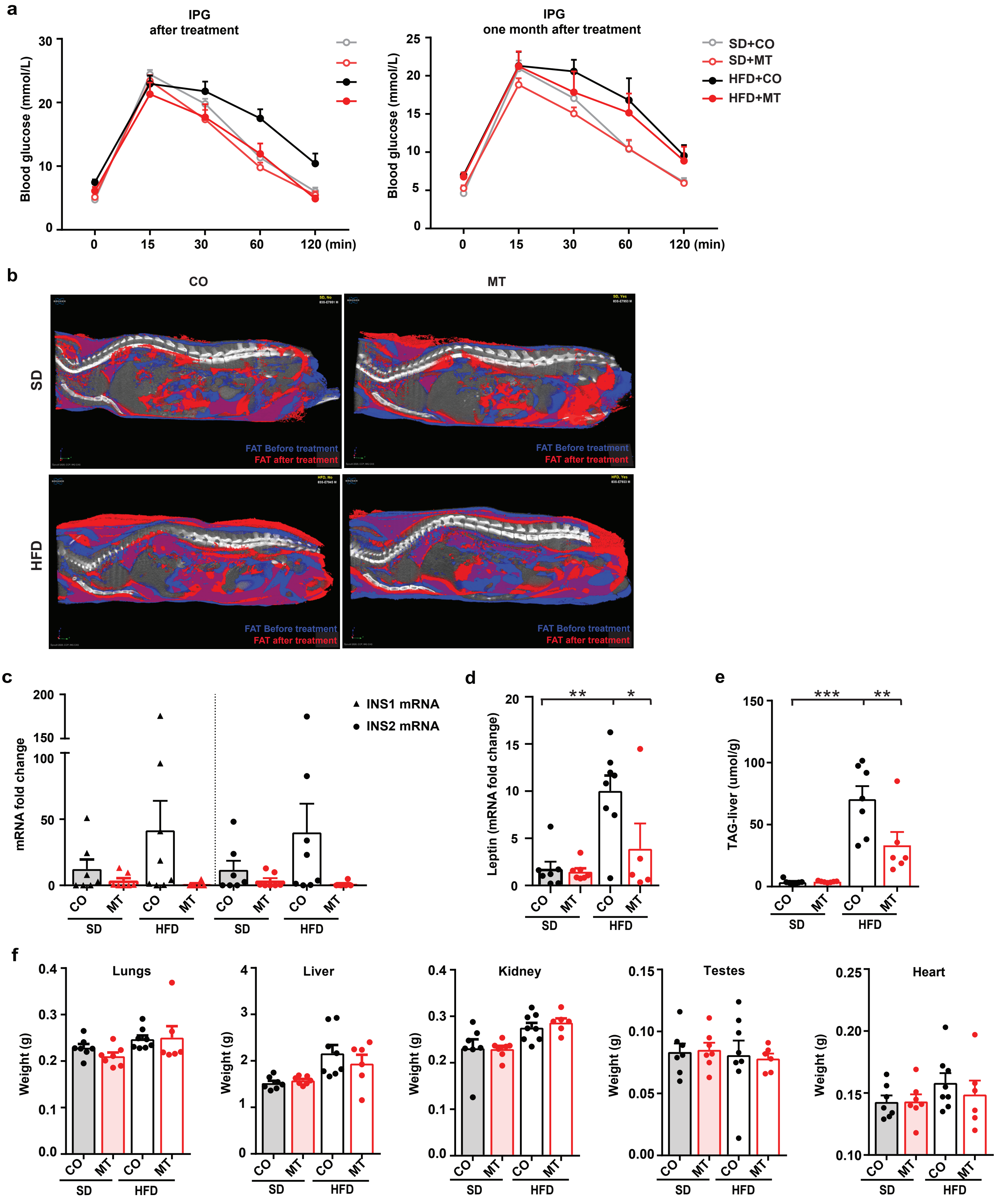

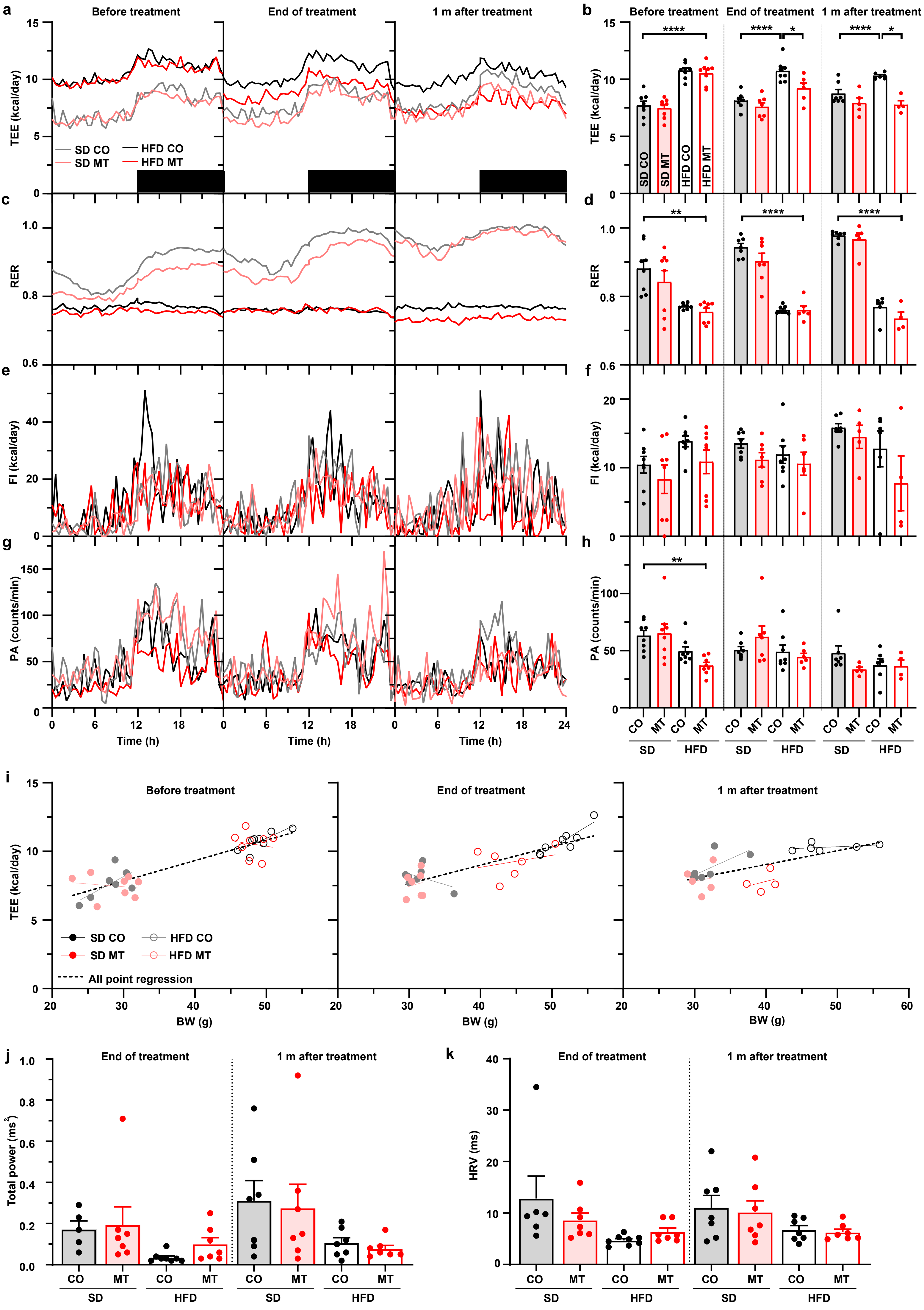

a
Before treatment
End of treatment
1 m after treatment
TEE (kcal/day)
SD CO
SD MT
HFD CO
HFD MT
b
Before treatment
End of treatment
1 m after treatment
c
RER
d
e
FI (kcal/day)
f
g
PA (counts/min)
Time (h)
h
CO
MT
SD
HFD
i
All point regression
BW (g)
j
Total power (ms²)
k
HRV (ms)

Supplementary Figure 6

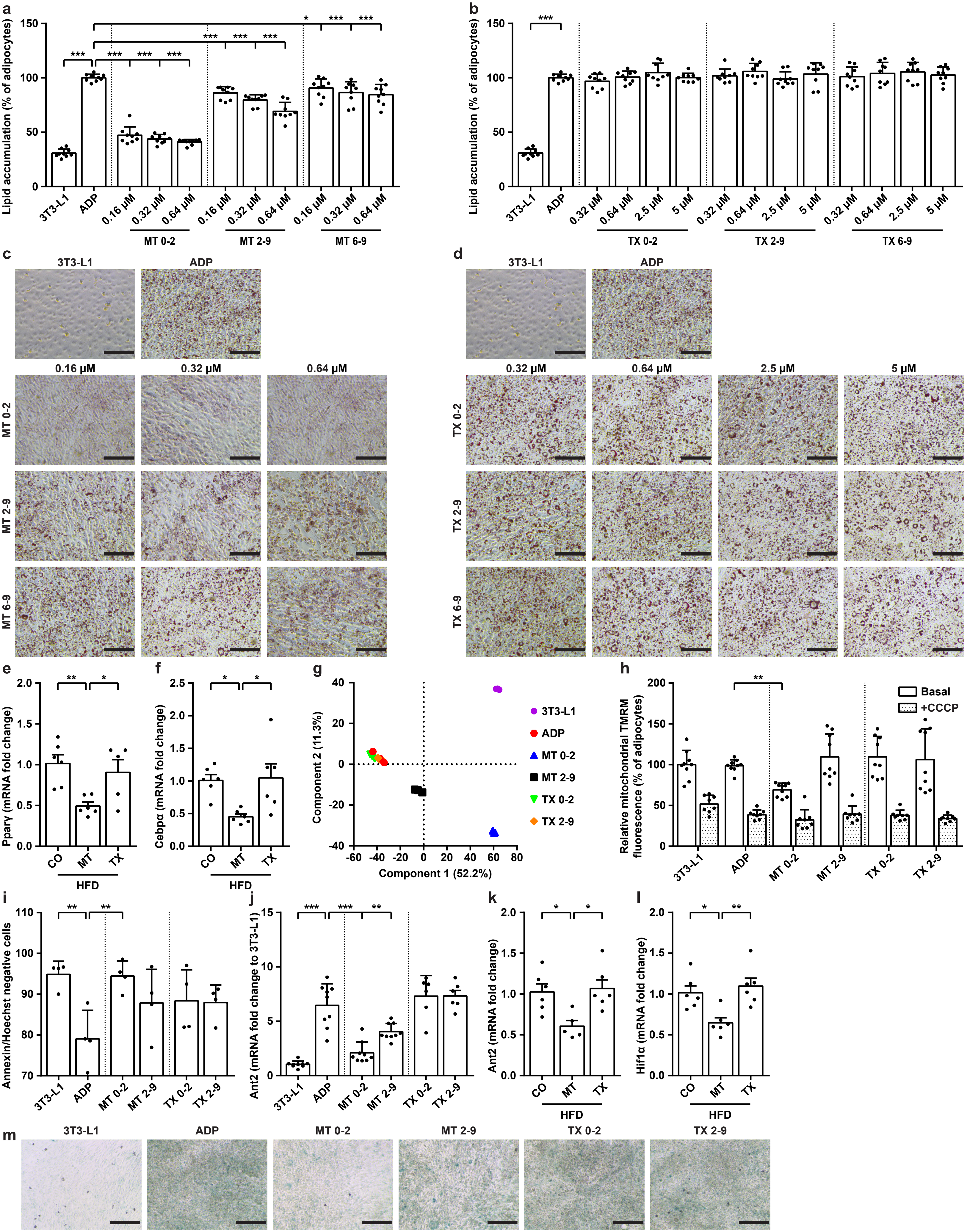

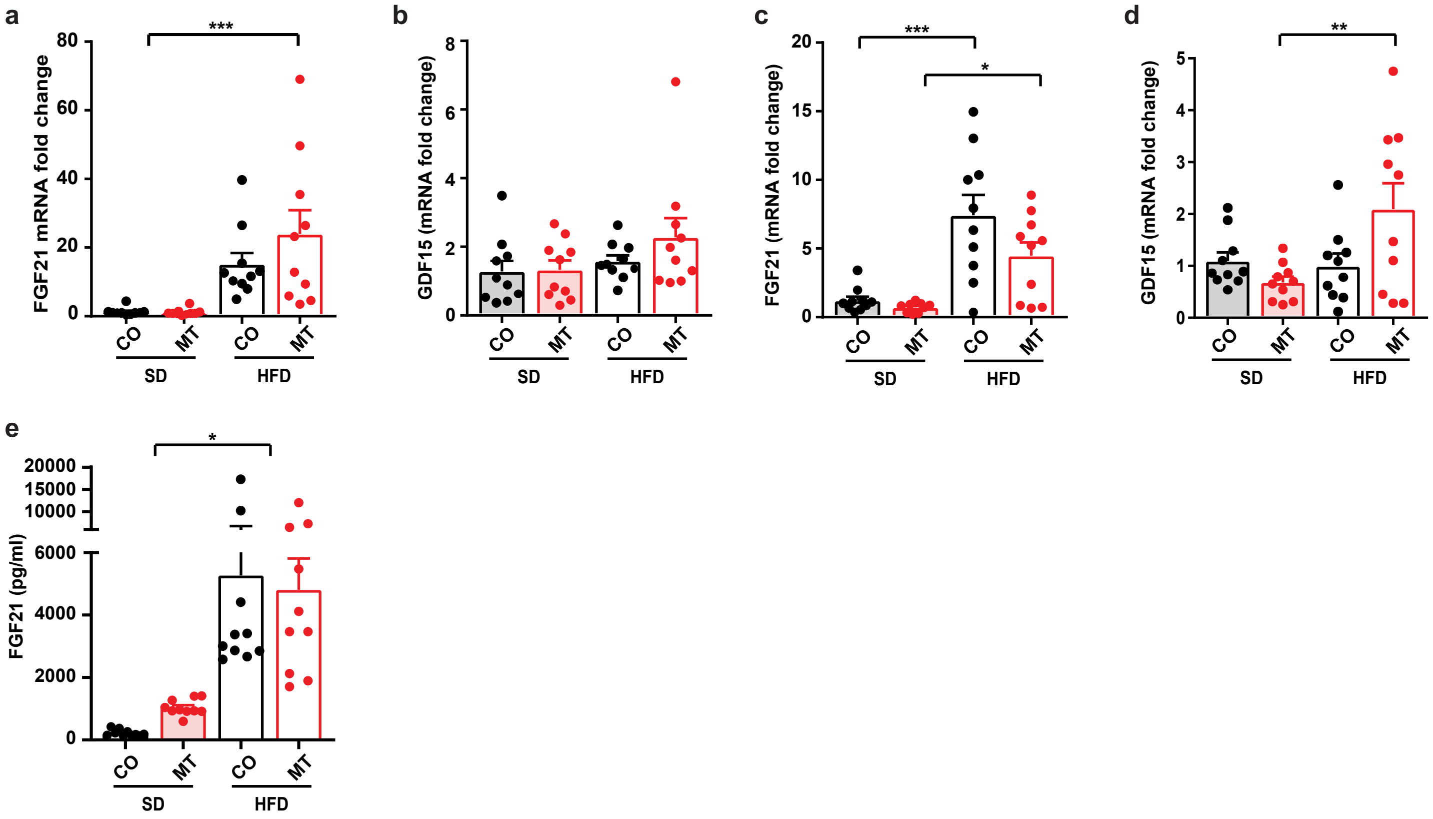
a
FGF21 mRNA fold change
80
60
40
20
0
***
CO
MT
CO
MT
SD
HFD
b
GDF15 (mRNA fold change)
8
6
4
2
0
c
FGF21 (mRNA fold change)
20
15
10
5
0
***
*
d
GDF15 (mRNA fold change)
5
4
3
2
1
0
**
e
FGF21 (pg/ml)
20000
15000
10000
6000
4000
2000
0
*
