# Supplementary material for: Mitochondrially targeted tamoxifen alleviates markers of obesity and type 2 diabetes mellitus in mice
Source: Nat Commun. 2022 Apr 6;13:1866. doi: 10.1038/s41467-022-29486-z (PMC8987092; doi:10.1038/s41467-022-29486-z)
Supplement: Supplementary file 3 — Reporting summary [file 41467_2022_29486_MOESM3_ESM.pdf]

nature portfolio

Corresponding author(s): Jiri Neuzil, Martin Haluzik, Sona Stemberkova Hubackova

Last updated by author(s): Nov 16, 2021

Reporting Summary

Nature Portfolio wishes to improve the reproducibility of the work that we publish. This form provides structure for consistency and transparency in reporting. For further information on Nature Portfolio policies, see our Editorial Policies and the Editorial Policy Checklist.

Statistics

For all statistical analyses, confirm that the following items are present in the figure legend, table legend, main text, or Methods section.

n/a	Confirmed	
☐	☒	The exact sample size (*n*) for each experimental group/condition, given as a discrete number and unit of measurement
☐	☒	A statement on whether measurements were taken from distinct samples or whether the same sample was measured repeatedly
☐	☒	The statistical test(s) used AND whether they are one- or two-sided<br>*Only common tests should be described solely by name; describe more complex techniques in the Methods section.*
☒	☐	A description of all covariates tested
☒	☐	A description of any assumptions or corrections, such as tests of normality and adjustment for multiple comparisons
☐	☒	A full description of the statistical parameters including central tendency (e.g. means) or other basic estimates (e.g. regression coefficient) AND variation (e.g. standard deviation) or associated estimates of uncertainty (e.g. confidence intervals)
☐	☒	For null hypothesis testing, the test statistic (e.g. *F*, *t*, *r*) with confidence intervals, effect sizes, degrees of freedom and *P* value noted<br>*Give P values as exact values whenever suitable.*
☒	☐	For Bayesian analysis, information on the choice of priors and Markov chain Monte Carlo settings
☒	☐	For hierarchical and complex designs, identification of the appropriate level for tests and full reporting of outcomes
☒	☐	Estimates of effect sizes (e.g. Cohen's *d*, Pearson's *r*), indicating how they were calculated

Our web collection on statistics for biologists contains articles on many of the points above.

Software and code

Policy information about availability of computer code

Data collection	Leica SP8 FLIM confocal microscope (Leica Microsystems), C1000™ Thermal Cycler (BioRad); LSR Fortessa instrument (Beckton Dickinson, Franklin Lakes, NJ, USA)
Data analysis	GraphPad Prism (version 8.4.3), GraphPad Prism 7.05v; FlowJo_V10, TTTR Data analysis v152, Leica Las X; ImageJ 1.52v software

For manuscripts utilizing custom algorithms or software that are central to the research but not yet described in published literature, software must be made available to editors and reviewers. We strongly encourage code deposition in a community repository (e.g. GitHub). See the Nature Portfolio guidelines for submitting code & software for further information.

Data

Policy information about availability of data

All manuscripts must include a data availability statement. This statement should provide the following information, where applicable:

- Accession codes, unique identifiers, or web links for publicly available datasets
- A description of any restrictions on data availability
- For clinical datasets or third party data, please ensure that the statement adheres to our policy

All the relevant data are supporting the findings of this study are available within this article or in the supplementary material. The datasets generated during and/or analysed during the current study are available from the corresponding author on reasonable request.

Field-specific reporting

Please select the one below that is the best fit for your research. If you are not sure, read the appropriate sections before making your selection.

☒ Life sciences ☐ Behavioural & social sciences ☐ Ecological, evolutionary & environmental sciences

For a reference copy of the document with all sections, see nature.com/documents/nr-reporting-summary-flat.pdf

Life sciences study design

All studies must disclose on these points even when the disclosure is negative.

Sample size	In animal studies we used 6-10 mice per group. The number of animals in each group is based on the smallest possible number of animals for the calculation of statistics, while adhering to the principles of the 3Rs (Replacement, Reduction and Refinement).
Data exclusions	~~No data were excluded from the analyses.~~
Replication	~~Main results from animal experiments were replicated in 4 different experiments. The cell culture results are from at least three independent experiments.~~
Randomization	Allocation of animals into groups were based on body weight and levels of glycemia.
Blinding	In this study blinding was not possible because treatment of animals were made by investigators as well as sample measurements.

Reporting for specific materials, systems and methods

We require information from authors about some types of materials, experimental systems and methods used in many studies. Here, indicate whether each material, system or method listed is relevant to your study. If you are not sure if a list item applies to your research, read the appropriate section before selecting a response.

Materials & experimental systems

n/a	Involved in the study	
☐	☒	Antibodies
☐	☒	Eukaryotic cell lines
☒	☐	Palaeontology and archaeology
☐	☒	Animals and other organisms
☐	☒	Human research participants
☐	☒	Clinical data
☒	☐	Dual use research of concern

Methods

n/a	Involved in the study	
☒	☐	ChIP-seq
☐	☒	Flow cytometry
☒	☐	MRI-based neuroimaging

Antibodies

Antibodies used	In our study we used primary rabbit anti-TOM20 antibody (1:100 in PBS, EPR15581-54, Abcam) and AlexaFluor 488 labeled secondary goat anti-rabbit antibody (1:1000 in PBS, A11034, ThermoFisher Scientific) for immunodetection of mitochondria on confocal microscope.
Validation	Primary antibody was tested without Alexa 488 labeled secondary antibody to avoid its nonspecific signal. To check its specific binding on mitochondria, colocalization with MitoTracker was tested.

Eukaryotic cell lines

Policy information about cell lines

Cell line source(s)	Mouse 3T3-L1 pre-adipocytes were acquired from ATCC.
Authentication	Cells have been authenticated by morphological analysis, as compared to the ATCC documentation. Similarly, development of adipocytes increasing senescence and lipid accumulation after cultivation of cells in special differentiation medium confirms the origin of cells.
Mycoplasma contamination	Cell line has been tested negative for mycoplasma contamination using Vero cells or MycoAlert Mycoplasma Detection Kit.
Commonly misidentified lines (See ICLAC register)	None

Animals and other organisms

Policy information about studies involving animals; ARRIVE guidelines recommended for reporting animal research

Laboratory animals

Aging experiment: Aged (18 months) and young (8 weeks) C57BL/6 mice; males+females
Obese and prediabetic experiment: C57BL/6J; 8 month old mice; males
Comparison of MitoTam and tamoxifen experiment: C57BL/6J mice; 16 months old; males
Prolonged effect of MitoTam experiment: C57BL/6 mice; 26 weeks old; males
Pair feeding experiment: C57BL/6J mice; 10 months old; males

Wild animals

Study did not involve wild animals.

Field-collected samples

~~All mice were maintained at 22 °C and 12 h/12 h light/dark regimen. At the end of the each experiment, animals were sacrificed between 9 am and 11 am by anesthesia and plasma/serum was separated from blood, and organs were collected and frozen at -80°C until further analysis.~~

Ethics oversight

Experiments were performed in agreement with the Animal Protection Law of the Czech Republic and were approved by the Ethics Committee of the Institute for Clinical and Experimental Medicine, Prague (permit number 41/2018) and Ethics Committee of the Institute of Molecular Genetics, Prague (permit number 51/2018).

Note that full information on the approval of the study protocol must also be provided in the manuscript.

Human research participants

Policy information about studies involving human research participants

Population characteristics

To obtain samples from lean subjects and patients with obesity (BMI ≥ 35 kg/m2) with or without type 2 diabetes patients of at least 50 years of age were recruited. Subcutaneous and visceral adipose tissue samples from lean subjects were taken during elective abdominal surgeries (cholecystectomy). Subcutaneous and visceral adipose tissue samples from patients with obesity with or without diabetes were taken during bariatric surgery (sleeve gastrectomy).
The MitoTam study was conducted on patients with advanced solid tumors. Assessment of glucose changes was not the primary aim of this study. However, the participation of several diabetic patients provided us with an unique opportunity to determine the effect of MitoTam on glucose level measured in panel of standard biochemical parameters for each patient.

Recruitment

Recruitment of patients for the obesity and diabetes study was organized by actively approaching potential study subject by clinical center of General University Hospital, Institute for Clinical and Experimental Medicine, Prague, Czech Republic as a part of ongoing studies (Research Project funded by Ministry of Health IKEM, IN 00023001 and RVO VFN64165).
~~Recruitment of patients for the MitoTam study was organized by actively approaching potential study subject by clinical center of General University Hospital, Prague, Czech Republic.~~

Ethics oversight

Written informed consent was signed by all participants prior to enrollment. The study was approved by the Human Ethics Review Board, First Faculty of Medicine and General University Hospital and Institute for Clinical and Experimental Medicine and Thomayer Hospital (approval No. G-18-51 and G-18-61), Prague, Czech Republic, and was performed in accordance with the guidelines proposed in the Declaration of Helsinki (2000) of the World Medical Association. The MitoTam study was performed in accordance with the National Institute for drug control (see Clinical data).

Note that full information on the approval of the study protocol must also be provided in the manuscript.

Clinical data

Policy information about clinical studies

All manuscripts should comply with the ICMJEguidelines for publication of clinical research and a completedCONSORT checklist must be included with all submissions.

Clinical trial registration

~~EudraCT 2017-004441-25~~

Study protocol

~~MitoTam-01~~

Data collection

~~Already finished, data cleared, database locked~~.

Outcomes

~~In Phase I the tolerability (safety) of the substance at different doses during the weekly administration of the investigational medicinal product was verified. Maximun tolerated dose and finding appropriate doses and safety criteria for phase Ib was determined. In phase Ib, the safety (frequency, severity and duration of adverse reactions) of doses proposed for repeated administration was assessed and a possible target group of malignances for MitoTam therapy was identified. Determination of changes in glucose level was not the aim of this trial. However, its measurement in the context of detecting changes in the patient´s biochemical parameters provided us with valuable information on the effect of MitoTam on metabolism. These data supported our experimental results.~~

Flow Cytometry

Plots

Confirm that:

- [x] The axis labels state the marker and fluorochrome used (e.g. CD4-FITC).
- [ ] The axis scales are clearly visible. Include numbers along axes only for bottom left plot of group (a 'group' is an analysis of identical markers).
- [ ] All plots are contour plots with outliers or pseudocolor plots.
- [ ] A numerical value for number of cells or percentage (with statistics) is provided.

Methodology

Sample preparation	Preparation of samples for flow cytometry is described in detail in the article (section Methods).
Instrument	Samples were measured by LSR Fortessa (Beckton Dickinson, Franklin Lakes, NJ, USA).
Software	The FlowJo_V10 software (Beckton Dickinson, Franklin Lakes, NJ, USA) was used.
Cell population abundance	Purity was determined by relevant staining and debriss was filtered out by the size using flow cytometry. 10,000 - 20,000 events were originally collected. Positively-gated cells showed approximatelly 98% purity.
Gating strategy	All samples were gated for FCSA and SSCA followed by FCSA and FCSH gating to select single cells. Cell death was expressed as the percentage of Hoechst positive/annexinV-647 positive population of cells.<br>To analyze mitochondrial potential using TMRM stainig, population of single cells (detected as described before) was then analyzed for TMRM signal using TexasRed channel.<br>All results are plotted as a bar graph from at last three independent experiments. Original plots are available from the corresponding author on reasonable request.

- [x] Tick this box to confirm that a figure exemplifying the gating strategy is provided in the Supplementary Information.
